# Proteomic analysis of proteins related to rice grain chalkiness using iTRAQ and a novel comparison system based on a notched-belly mutant with white-belly

**DOI:** 10.1186/1471-2229-14-163

**Published:** 2014-06-12

**Authors:** Zhaomiao Lin, Xincheng Zhang, Xiaoyu Yang, Ganghua Li, She Tang, Shaohua Wang, Yanfeng Ding, Zhenghui Liu

**Affiliations:** 1 College of Agronomy, Nanjing Agricultural University/Key Laboratory of Crop Physiology Ecology and Production Management, Ministry of Agriculture, Nanjing 210095, PR China; 2 Jiangsu Collaborative Innovation Center for Modern Crop Production, Nanjing 210095, PR China

**Keywords:** Rice, Grain chalkiness, iTRAQ, White-belly, Notched-belly mutants

## Abstract

**Background:**

Grain chalkiness is a complex trait adversely affecting appearance and milling quality, and therefore has been one of principal targets for rice improvement. Eliminating chalkiness from rice has been a daunting task due to the complex interaction between genotype and environment and the lack of molecular markers. In addition, the molecular mechanisms underlying grain chalkiness formation are still imperfectly understood.

**Results:**

We identified a notched-belly mutant (DY1102) with high percentage of white-belly, which only occurs in the bottom part proximal to the embryo. Using this mutant, a novel comparison system that can minimize the effect of genetic background and growing environment was developed. An iTRAQ-based comparative display of the proteins between the bottom chalky part and the upper translucent part of grains of DY1102 was performed. A total of 113 proteins responsible for chalkiness formation was identified. Among them, 70 proteins are up-regulated and 43 down-regulated. Approximately half of these differentially expressed proteins involved in central metabolic or regulatory pathways including carbohydrate metabolism (especially cell wall synthesis) and protein synthesis, folding and degradation, providing proteomic confirmation of the notion that chalkiness formation involves diverse but delicately regulated pathways. Protein metabolism was the most abundant category, accounting for 27.4% of the total differentially expressed proteins. In addition, down regulation of PDIL 2–3 and BiP was detected in the chalky tissue, indicating the important role of protein metabolism in grain chalkiness formation.

**Conclusions:**

Using this novel comparison system, our comprehensive survey of endosperm proteomics in the notched-belly mutant provides a valuable proteomic resource for the characterization of pathways contributing to chalkiness formation at molecular and biochemical levels.

## Background

Chalkiness is the opaque part of rice grain. Chalky grains have a lower density of starch granules compared to vitreous ones, and are more prone to breakage during milling. It is also detracts from the visual appearance, adversely affecting consumer acceptability and lowering the overall market value [1]. In many rice-producing areas, high ratio of chalky grains is a major concern that decreases grain quality. For China as an example, many early-season indica and japonica varieties are of high grain chalkiness [2]. Therefore, eliminating chalkiness from rice grain has been one of the prime goals in rice breeding.

High temperatures as well as the combination of high relative humidity and low vapor pressure deficit during grain filling has been shown as the most important environmental conditions affecting grain chalkiness [3]. Current grain filling temperatures are already approaching critical levels in many rice growing counties, resulting in high occurrence of grain chalkiness, currently a major problem for rice production in Asian countries [4]. Therefore, understanding of the mechanisms of grain chalking is indispensable to develop a strategy for reducing the high rate of chalky grains under the likely scenario of global warming.

Grain chalkiness is a complex trait controlled by quantitative trait loci (QTLs). More than 40 QTLs contributing to the percentages of chalky grains and degrees of endosperm chalkiness have been mapped in the rice genome using F2 segregation populations, chromosome segment substitution lines, or introgression lines [2]. These QTLs distributed across all the 12 rice chromosomes, among which *qPGWC-7* and *qPGWC-8* have been fine-mapped [2]. However, only few QTLs have been isolated and functionally analyzed, and few genes have been identified [5]. So far, the molecular mechanisms underlying the formation of rice grain endosperm chalkiness still remain poorly understood.

In rice grains, starch is the predominant storage substance, constituting nearly 90% of the total dry mass. Microscopic observation showed that starch granules of the chalky endosperm were loosely packed as compared with those of the translucent part [6]. Thus, the incomplete accumulation of starch has been considered as the main cause for chalkiness formation. Most of studies concerning grain chalkiness have been focused on the genes encoding enzymes involved in starch synthesis or carbon metabolism, such as granule-bound sucrose synthase, pyruvate orthophosphate dikinase, starch-branching enzyme IIb, and debranching enzymes as reviewed by Liu *et al.*[5].

On the other hand, the role of the other major component of rice endosperm, the proteins, has been underestimated to some extent. Recent data report that other diverse factors especially the proteins are also associated with the opaque phenotype. For example, overexpression of a rice binding protein (BiP) produced an opaque phenotype seed with a floury endosperm [7]. In addition, Lin *et al.*[8] investigated the effects of high temperature during grain filling on the expression of storage proteins, and found that the chalky grains contained reduced amount of prolamins, implying the relationship between prolamins and chalky structure in rice grains. Collectively, these studies indicate the potential role of protein synthesis or nitrogen metabolism in the formation of grain chalkiness, which needs further investigation.

**Figure 1:**
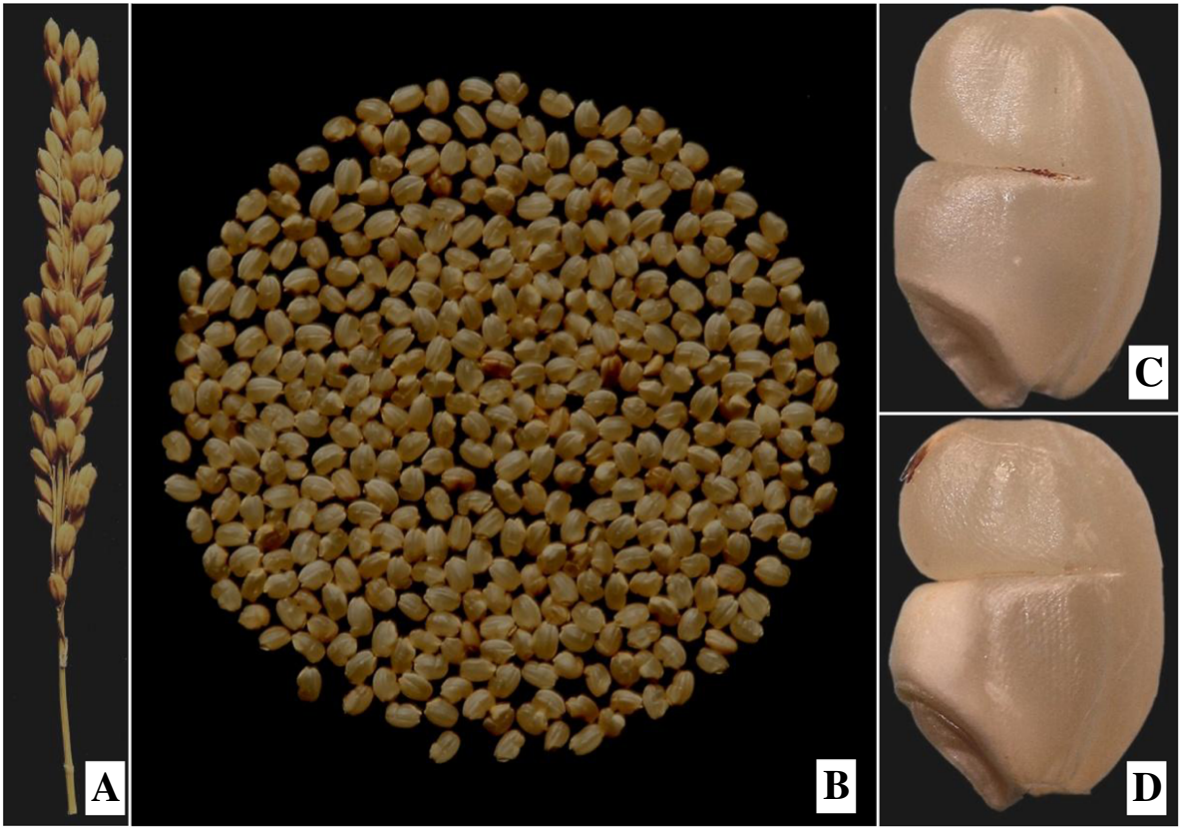
**Mutant of notched-belly with white-belly near embryo (DY1102). A**, The panicle of DY1102; **B**, Brown rice of DY1102; **C**, Notched-belly grain without white-belly; **D**, Notched-belly grain with white-belly in bottom half part proximal to the embryo.

Proteomics is an effective strategy for directly and globally investigating the protein expression patterns and its respective posttranslational modifications. It has been employed in the studies of grain chalkiness under high temperature [8]. Lin *et al.*[9] analyzed heat stress response of several different cultivars including high-chalky types, and found that sHSP was positively correlated with the appearance of chalky kernels. Li *et al.*[10] reported that accumulation of isoforms of PPDK and pullulanase was prone to high night temperature. However, comparative display of protein expression patterns in these studies were performed either between varieties [9] or between grains under different treatments for a given variety [8,10], which can not avoid the effect of genotype and environment.In this study, a notched-belly mutant with white-belly (DY1102) was used as material, which has high percentage of notched-belly grain. Interestingly, the white-belly only occurs in the bottom part proximal to the embryo, with the upper half part being translucent (Figure 1). Based on this mutant, we developed a novel comparison system that can minimize the influence of genetic backgrounds and growing environment. A comparative proteomic analysis by iTRAQ (isobaric tags for relative and absolute quantification) resulted in detection and identification of a total of 113 proteins responsible for grain chalkiness. Our comprehensive and deep survey of endosperm proteomics in the notched-belly mutants with white-belly should provide an excellent starting material for further elucidating the molecular and biochemical basis of rice grain chalkiness.

## Methods

### Plant materials

Wuyujing3 is a benchmark japonica rice variety of China for its high eating quality, while it has weak points such as relatively lower yield potential, susceptibility to rice stripe virus, and high chalky grain rate (usually being above 30%) [6]. In 2007, 2000 g seeds of Wuyujing3 were treated with 0.5% ethyl methane sulfonate (EMS) for 16 hours at room temperature (about 25°C). Before sowing, seeds were soaked for 24 hours and then drained 24 hours. M_1_ seeds was grown in field and mixed harvested. Selection on grain chalkiness was performed at M_3_ seeds after harvest of M_2_ plants in 2009. A notched-belly mutant with white-belly (DY1102) was identified after harvest of 2010 (M_4_ seeds). This mutant has high percentage of notched-belly grain, being 83.36% in 2011 (M_5_ seeds) and in 73.74% in 2012 (M_6_ seeds; Table 1). It is interesting that major part of the notched-belly grains have white-belly, which is visible on the fifth day after anthesis. This is obvious for the grains on the middle primary rachis of the panicle, which was used as the sampling position within panicle in this study (Table 1). Moreover, the white-belly only occurs in the bottom half part proximal to the embryo, i.e. below the notched line (Figure 1).

**Table 1:** Positional variation within panicle in ratios of white-belly grains to notched-belly grains in 2011 and 2012

**Grain position**	**Notched-belly grain/total grain (%)**		**White-belly grain/notched-belly grain (%)**	
	**2011**	**2012**	**2011**	**2012**
TPR	88.73	68.48	85.66	72.22
TSR	85.82	72.38	62.61	76.32
MPR	84.09	68.79	83.33	84.03
MSR	79.38	81.07	58.01	73.72
BPR	81.00	72.49	69.55	82.48
BSR	82.08	84.44	50.57	71.05
TWP	83.36	73.74	71.19	77.05

Note: TPR, Top primary rachis; TSR, top secondary rachis; MPR, middle primary rachis; MSR, middle secondary rachis; BPR, bottom primary rachis; BSR, bottom secondary rachis; TWP, the whole panicle.

### Comparison system

**Figure 2:**
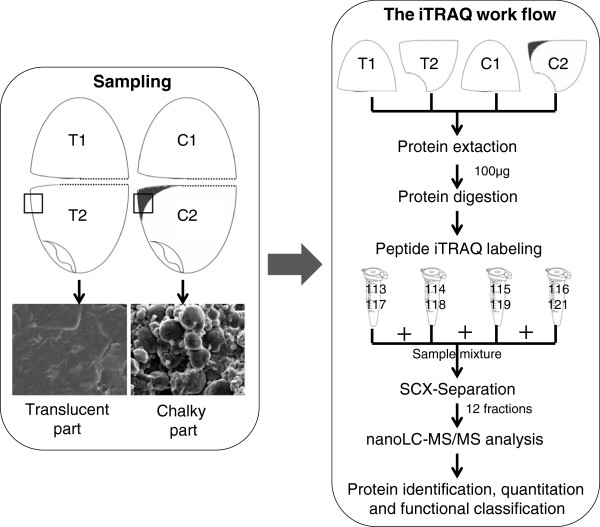
**The experimental scheme of the sampling and iTRAQ analysis.** C1 and C2, the upper and bottom half part of grain with white-belly, respectively. T1 and T2, the upper and bottom half part of grain without white-belly, respectively. Note that the embryos of C2 and T2 were removed.

Using DY1102, we developed a novel comparison system aiming to evaluate the effect of chalkiness on the protein expression. As shown in Figure 2, notched-belly grains without white-belly was used as the control. First, comparison between the bottom part (T2) and upper part (T1) can quantify the effect of embryo. Second, comparison of the bottom part (C2) and upper part (C1) of grain with white-belly can evaluate the compound effect of embryo and chalkiness. Thus, the effect of chalkiness can be measured through a further comparison between C2 vs C1 and T2 vs T1, by which the embryo effect was minimized. Importantly, these comparisons are made within the same grain, which has the identical genotype or genetic background, and thus are more efficient than those made between different grains within one panicle or from different varieties.

### SEM and amylopectin structure analysis

At maturity, grains from the middle primary rachis of the panicle were collected and then dehusked as brown rice. Typical notched-belly grains with and without white-belly were selected. After the embryo removed, grains were cut into two parts along the notched line, resulting in two kinds of samples for both grain types, i.e. T1 and T2 for the translucent grains and C1 and C2 for the grains with white-belly (Figure 2). For scanning electron microscope (SEM) analysis, samples were completely dried under low pressure and then were transversely cut with a razor blade, producing a fracture rather than a clean cut. The fracture was sputter-coated with gold in vacuum and observed by SEM (Hitachi S-3000 N) at an accelerating voltage of 15 kV.

Amylopectin of the four samples were extracted and purified using the alkaline-steeping method of Patindol *et al.*[11]. Amylopectin was debranched by isoamylase (I5284-5MU, Sigma-Aldrich) and then separated by high-performance size-exclusion chromatography, according to Yang *et al.*[12]. The degree of polymerization (DP) of the linear fractions in debranched amylopectin was calculated as their molecular weight divided by 162. The analyses were performed with three replications for each sample.

### Protein extraction

Proteins of the four parts were extracted according to the method of Ding *et al.*[13]. Briefly, samples were grinded about 0.1 g were suspended with ice-cold 10% (w/v) trichloroacetic acid in acetone containing 10 mM dithiothreitol and 1 mM phenylmethylsulfonyl fluoride, and then incubated at −20°C for 1 h, followed by centrifugation at 20000 × g for 30 min at 4°C in a refrigerated high-speed centrifuge. The precipitate was washed three times by suspending in cold acetone. After vacuum drying, the precipitate (~40 mg) was dissolved in 1 ml 0.5 M triethylammonium bicarbonate buffer. Protein concentration was measured by the Bradford method using bovine serum albumin as the standard.

### In-solution trypsin digestion and iTRAQ procedure

Trypsin digestion and iTRAQ analysis were performed by Beijing Genomics Institute. Total protein (~100 μg) of each sample was reduced by adding dithiothreitol to a final concentration of 10 mM and incubated for 1 h, then alkylated with 55 mM iodoacetamide for 1 h at room temperature. Samples were digested using sequencing grade trypsin solution (10 ng/μl in 25 mM ammonium bicarbonate) for 12 h at 37°C. After digestion, the peptide segment was freeze-dried in a centrifugal speed vacuum concentrator.

Two replications for each sample were performed for iTRAQ analysis. The peptide segment of each sample was labeled using iTRAQ 8-plex kits according to the manufacturer's manual (AB Sciex Inc., USA). As shown in Figure 2, two replications of T1 were labeled with reagent 113 and 117, T2 with reagent 114 and 118, C1 with reagent 115 and 119, and C2 with reagent 116 and 121, respectively. After labeling and quenching, samples were combined and lyophilized before redissolving in 4 ml of buffer A (25 mM NaH_2_PO_4_ in 25% acetonitrile, pH 2.7). Fractionation was conducted though the strong cation exchange chromatography on an Ultremex SCX column using HPLC (Shimadzu LC-20AB). The mixed sample was eluted with buffer A for 10 min, then eluted with a gradient of 5%-80% buffer B (25 mM NaH_2_PO_4_, 1 M KCl in 25% acetonitrile, pH2.7) for 12 min, at a flow rate of 1 ml/min. The process was monitored by absorbance at 214 nm simultaneously. The resulting 12 fractions were collected according to the peak area, and then were desalted with StrataX desalting column and lyophilized.

### LC-MS/MS analysis

The system of UPLC (nanoACQuity, Waters) coupled with tandem mass spectrometry (TripleTOF 5600, AB SCIEX, Concord, ON) was used for peptides identification and quantification. The data acquisition was performed according to Wang *et al.*[14]. Briefly, a TripleTOF 5600 System fitted with a Nanospray III source and a pulled quartz tip as the emitter was used for tandem mass spectrometry. Data was acquired using an ion spray voltage of 2.5 kV. The MS was operated with TOF-MS scans. For information dependant acquisition, the MS survey scans were acquired in 250 ms and the top 30 product ion were selected for MS/MS scans.

### Bioinformatics analysis

All MS/MS spectra were searched against in the NCBInr Oryza sativa sequence databases and UniProtKB/Swiss-Prot database with MASCOT software (Matrix Science, UK; version 2.3.02). For biological replications, spectra from the 12 fractions were combined into one file and searched. The search parameters were as follows: (1) allowing one missed cleavage in the protein trypsin digests; (2) fixed modifications of carbamidomethylation at Cys, variable modifications of oxidation at Met and iTRAQ 8-plex at Tyr; (3) peptide tolerance was set at ±0.1 Da and MS/MS tolerance was set at ±0.05 Da. The peptide charge was set as Mr, and monoisotopic mass was chosen. The iTRAQ 8-plex was chosen for quantification during the search.

The search results were filtered before data exportation. The filters were used for protein identification with these options: significance threshold P < 0.05 (with 95% confidence) and ion score or expected cutoff less than 0.05 (with 95% confidence). For protein quantitation, the filters were set as follows: (1) “median” was chosen for the protein ratio type; (2) the minimum precursor charge was set to 2 and minimum peptides were set to 2, and only unique peptides were used for quantitation; and (3) normalization by median intensities, and outliers were removed automatically. The peptide threshold was set as above for identity. A 1.2-fold change was set to identify significant differentially expressed proteins in addition with *P*-value < 0.05.

The differentially expressed proteins were classified using the Gene Ontology database and Kyoto Encyclopedia of Genes and Genomes database.

## Results

### Morphological and amylopectin structure of the chalky endosperm

**Figure 3:**
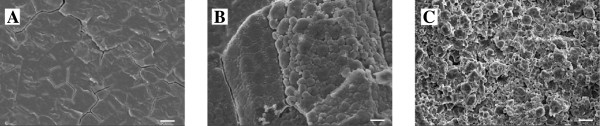
**Scanning electron microscopy images of starch granule in DY1102 and WT (wide type). A**, The translucent tissue of the upper half part (above the notched line) of grains with white-belly; **B**, The white-belly tissue of grains from wild type, Wuyujing3; **C**, The chalky tissue of the bottom half part (below the notched line) of grains with white-belly. Bars = 100 μm.

SEM photos show the typical characteristic of the chalky tissues of the grains with white-belly (Figure 3). Starch granules are loosely packed in the opaque endosperm cells (Figure 3C), as compared with that of the translucent tissues of grains without white-belly or the translucent upper part of the grains with white-belly (Figure 3A). In addition, in comparison with that of the wild type (Figure 3B), chalky tissues of DY1102 are easily broken under the mechanical stress during sample preparation, indicating differences in chemical composition or microstructure of the chalky tissue between DY1102 and its wild type.

**Figure 4:**
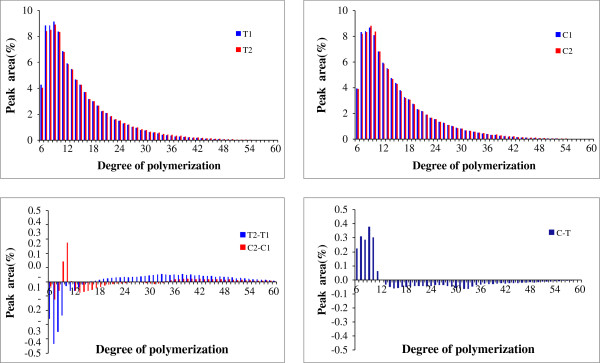
The chain-length distribution of amylopectin of the chalky and translucent parts grains with white-belly (C) and without white-belly (T).

In this study, notched-belly grain without white-belly was used as the control. Adopting the above mentioned comparison system in the Methods section, the effect of chalkiness on starch fine structure can be evaluated by the comparison between C2 vs C1 and T2 vs T1. The result revealed that the chalky tissue contained more short chain (DP ≤ 12) and less medium and long chain as compared with the translucent tissue (Figure 4). This finding agrees with that of Liu *et al.*[5] and Patindol and Wang [15].

### Proteins identified by iTRAQ and their functional classification

Total proteins in the four samples (T1, T2, C1, and C2) were explored using the iTRAQ technique with two biological replications. Analysis of protein extracts from these samples resulted in the detection and identification of 1503 (4031 unique peptides; Additional file 1). The number of rice endosperm proteins identified in this study is larger than that using 2-DE, being about 500 spots by Li *et al*. [10] and 302 by Yang *et al.*[16]. It is greater than that obtained by multidimensional protein identification technology, being 822 proteins as reported by Koller *et al.*[17].

**Figure 5:**
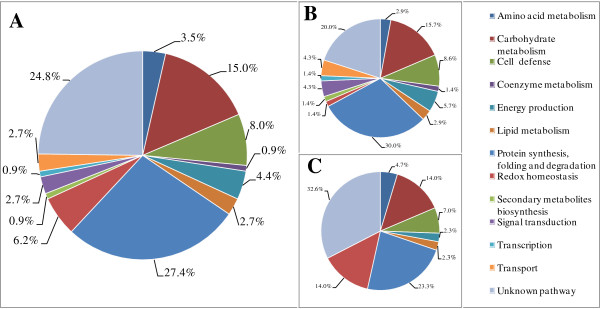
**Functional classification and distribution of the differentially expressed proteins via the Gene Ontology (GO) database. A**, Total differentially expressed proteins(113); **B**, Up-regulated proteins (70); **C**, Down-regulated proteins (43).

Proteins were considered as being differentially expressed according to Neilsen *et al.*[18], which have both a fold-change more than 20% and a p-value below 0.05. A total of 113 proteins showed differential expression by comparison between C1 vs C2 and T1 vs T2, i.e. the proteins responsible for chalkiness formation (Tables 2 and 3). Among them, 70 proteins were up-regulated and 43 down-regulated. According to Gene Ontology (GO) database, these proteins fell into 13 major categories. As shown in Figure 5, the most abundant category was classified as being involved in protein synthesis, folding and degradation (27.4% of the total differentially expressed proteins). The second most abundant class consisted of proteins with yet unidentified function or proteins with no detectable homology to other predicted proteins in the database (24.8% of the total differentially expressed proteins). The third abundant category belongs to carbohydrate metabolism, accounting for 15.0% of the total differentially expressed proteins. Furthermore, seven proteins associated with redox homeostasis have been detected.

**Table 2:** Functional classification of up-regulated proteins

**Accession**	**Protein Name**	**Score**	**Mass (kDa)**	**Cov. (%)**	**Unique peptide**	**C2/C1**	**T2/T1**	**C/T**
Amino acid metabolism								
gi|385717668	5-methyltetrahydropteroyltriglutamate-homocysteine methyltransferase	768	100.7	12.1	7	0.322	0.260	1.238
gi|77548611	2-isopropylmalate synthase A	677	78.0	23	9	0.938	0.721	1.301
Carbohydrate metabolism								
gi|78099751	Fructose-bisphosphate aldolase cytoplasmic isozyme	921	48.7	24	4	0.855	0.662	1.292
gi|113632010	Triosephosphate isomerase, chloroplastic	315	38.8	20.4	4	0.400	0.290	1.379
gi|113548195	Probable rhamnose biosynthetic enzyme 1	241	91.2	7.3	3	0.382	0.261	1.464
gi|40253646	putative starch synthase DULL1	155	231.2	3	5	1.451	0.990	1.466
gi|113580063	UDP-glucose 4-epimerase GEPI48	183	45.8	6.8	2	0.312	0.208	1.500
gi|113548194	UDP-glucuronic acid decarboxylase 1	220	46.1	14.6	3	0.566	0.340	1.665
gi|222628767	2-oxoglutarate dehydrogenase, mitochondrial	249	129.0	4.9	4	0.163	0.096	1.698
gi|113549799	UDP-glucose 6-dehydrogenase	188	65.0	11.9	3	0.173	0.081	2.136
gi|75327567	Probable cinnamyl alcohol dehydrogenase 6	92	46.0	4.2	1	0.437	0.135	3.237
gi|75133690	UDP-arabinopyranose mutase 3	243	49.9	16.7	5	0.270	0.074	3.649
gi|75156168	Hexokinase-1	152	56.2	4.6	2	0.333	0.054	6.167
Cell defense								
gi|113533098	Heterogeneous nuclear ribonucleoprotein A2/B1-like	131	50.1	10.5	2	1.567	1.241	1.263
gi|113580000	Late embryogenesis abundant protein Lea14-A	63	20.4	7.3	1	1.528	1.128	1.355
gi|22711545	Putative cytochrome P450	58	120.8	0.9	1	0.088	0.051	1.725
gi|113649843	P21 protein, putative, expressed	269	29.3	21.8	5	1.632	0.824	1.981
gi|113649501	Pathogenesis-related protein 1	197	21.3	38.1	4	1.380	0.500	2.760
gi|113649505	Major pollen allergen Bet v 1-D/H	1402	21.5	75.3	10	1.814	0.567	3.199
Coenzyme metabolism								
gi|54291831	C-1-tetrahydrofolate synthase, cytoplasmic	57	36.7	4.8	1	0.407	0.159	2.560
Energy production								
gi|113548489	ATP-dependent transporter ycf16, putative, expressed	172	38.0	15.1	3	0.900	0.747	1.205
gi|75294330	Probable succinyl-CoA ligase [ADP-forming] subunit alpha, mitochondrial	205	41.3	12.1	3	0.998	0.645	1.547
gi|113535585	Putative vacuolar proton-ATPase	475	79.8	14	2	0.400	0.110	3.636
gi|113533574	Putative vacuolar ATPase B subunit	1023	60.4	30.9	9	0.304	0.082	3.707
Lipid metabolism								
gi|113595159	Sterol carrier protein 2-like	210	18.4	28.7	3	1.084	0.820	1.322
gi|122248693	Non-specific lipid-transfer protein 3	241	14.1	24.8	2	1.598	1.122	1.424
Protein synthesis, folding and degradation								
gi|113579500	Protein translation factor SUI1 homolog	63	16.8	11.3	1	0.977	0.797	1.226
gi|122246932	Cysteine proteinase inhibitor 8	216	13.5	36.6	2	1.247	0.995	1.253
gi|38605801	40S ribosomal protein S27	288	7.4	66.7	2	0.855	0.672	1.272
gi|125584147	Tubulin-specific chaperone A	67	17.2	8	1	1.801	1.404	1.283
gi|108711183	26S proteasome non-ATPase regulatory subunit 10, putative, expressed	259	29.4	25.4	4	0.936	0.719	1.302
gi|113535779	26S proteasome regulatory particle triple-A ATPase subunit 6	177	58.0	18.6	5	0.437	0.326	1.340
gi|1703380	ADP-ribosylation factor 2	336	24.4	35.4	5	0.623	0.463	1.346
gi|222641792	Heat shock protein 81-2	461	56.5	19.5	2	1.535	1.110	1.383
gi|113531460	Bowman-Birk type wound-induced proteinase inhibitor WIP1	167	15.2	10	1	1.353	0.946	1.430
gi|113649350	Eukaryotic translation initiation factor 5A-2	212	22.6	29.4	3	1.179	0.821	1.436
gi|730456	40S ribosomal protein S19	375	20.1	41.1	6	0.860	0.581	1.480
gi|391875	40S ribosomal protein S20	135	17.7	21.4	1	0.847	0.562	1.507
gi|113550094	T-complex protein 1 subunit theta	244	70.8	11.7	4	0.367	0.240	1.529
gi|14589381	Alanine--tRNA ligase	228	95.3	9.9	5	0.797	0.474	1.681
gi|122232855	Clathrin heavy chain 1	229	224.0	6	7	0.490	0.274	1.788
gi|62900360	Importin subunit alpha-1a	159	67.0	5.3	2	0.290	0.156	1.859
gi|255672966	Putative density regulated protein drp1	244	28.6	38.2	4	1.035	0.547	1.892
gi|122169274	Coatomer subunit delta-2	93	69.0	7.3	3	0.521	0.220	2.368
gi|113548306	40S ribosomal protein S7	90	28.9	16.1	2	0.450	0.173	2.601
gi|75325389	Eukaryotic initiation factor 4A-3	428	53.5	25.1	8	0.514	0.173	2.971
gi|38347158	Chaperone protein ClpC1, chloroplastic	294	116.5	12.5	6	0.986	0.295	3.342
Redox homeostasis								
gi|122166938	Peroxidase 2	172	35.5	13.1	3	1.418	1.045	1.357
Secondary metabolites biosynthesis								
gi|75261413	4-hydroxy-3-methylbut-2-en-1-yl diphosphate synthase, chloroplastic	180	93.3	6	3	0.167	0.027	6.185
Signal transduction								
gi|122235035	Calmodulin-3	276	20.2	22.8	1	1.310	1.083	1.210
gi|75323484	Calmodulin-2	510	20.5	22.8	1	1.434	1.180	1.215
gi|113532001	Ras-related protein Rab7	136	28.0	16.9	3	0.692	0.390	1.774
Transcription								
gi|113622935	Putative quinone-oxidoreductase QR2	199	26.1	25.1	4	0.625	0.478	1.308
Transport								
gi|113639089	Protein CutA1, chloroplastic	348	21.6	22.9	3	1.327	1.045	1.270
gi|113649744	Sulfurtransferase	461	47.3	32.8	8	0.977	0.680	1.437
gi|113578690	ADP, ATP carrier protein 1, mitochondrial	197	50.6	9.2	3	0.502	0.137	3.664
Unknown pathway								
gi|113549649	hypothetical protein	166	12.0	17.4	2	1.777	1.423	1.249
gi|113579243	Pleckstrin homology domain-containing protein 1	129	21.9	7	1	0.916	0.728	1.258
gi|113596594	Os06g0711900	105	17.7	11.2	1	1.618	1.267	1.277
gi|3492928	16 kDa oleosin isoform R16	131	17.7	6.1	1	1.363	1.031	1.322
gi|113623942	Os08g0459300	204	27.3	29.2	3	1.222	0.920	1.328
gi|113596416	Putative phytocyanin protein, PUP2	158	28.4	12.2	3	1.217	0.908	1.340
gi|113532536	Outer-envelope membrane of Chloroplasts 34-like	425	747.7	2.5	10	1.210	0.871	1.389
gi|77550956	EF hand family protein	164	12.1	10.4	1	1.423	1.021	1.394
gi|75106519	Early nodulin-like protein 1	512	26.8	12.7	2	1.577	1.096	1.439
gi|53792234	Bowman-Birk type bran trypsin inhibitor	77	33.2	4.5	1	1.109	0.752	1.475
gi|46981317	unknown protein	70	60.7	4.5	2	0.636	0.380	1.674
gi|113547324	Gamma-thionin PPT, putative, expressed	573	10.7	27.2	2	1.552	0.920	1.687
gi|113623527	Os08g0327700 (Putative seed maturation protein)	224	17.6	13.3	1	1.792	0.965	1.857
gi|113578614	Putative pentatricopeptide repeat-containing protein At1g10330	51	58.4	1.4	1	1.884	0.516	3.651

Note: Notched-belly grain without white-belly was used as the control. The effect of embryo was quantified by comparison between the bottom part (T2) and upper part (T1). Comparison of the bottom part (C2) and upper part (C1) of grain with white-belly indicated the compound effect of embryo and chalkiness. Therefore, the effect of chalkiness can be evaluated through a further comparison between C2 vs C1 and T2 vs T1, by which the embryo effect was eliminated. Cov. , average percentage of assigned peptides to the predicted protein.

**Table 3:** Functional classification of down-regulated proteins

**Accession**	**Protein Name**	**Score**	**Mass (kDa)**	**Cov. (%)**	**Unique peptide**	**C2/C1**	**T2/T1**	**C/T**
Amino acid metabolism								
gi|584706	Aspartate aminotransferase, cytoplasmic	320	49.8	15.7	6	0.788	1.150	0.685
gi|113535557	Putative glycine decarboxylase complex H-protein	117	20.8	15.2	2	1.055	1.308	0.807
Carbohydrate metabolism								
gi|222640045	Putative aconitate hydratase, cytoplasmic	926	120.0	9.6	6	0.208	0.821	0.253
gi|28190676	Transketolase, chloroplastic	221	92.7	9.2	4	0.065	0.137	0.474
gi|113534863	Putative xylanase inhibitor	123	49.4	8.8	3	0.637	0.872	0.731
gi|108885236	Glucose and ribitol dehydrogenase homolog	276	37.3	23.7	6	1.015	1.288	0.788
gi|75114635	Probable alpha-glucosidase	676	102.6	5.1	4	1.135	1.364	0.832
gi|108707955	2,3-bisphosphoglycerate-independent phosphoglycerate mutase (PGAM)	261	72.8	6.1	1	0.040	-	-
Cell defense								
gi|75298023	17.9 kDa class I heat shock protein	301	22.5	17.4	2	1.147	1.756	0.653
gi|1169521	Embryonic abundant protein 1	151	12.3	35.8	3	1.792	2.271	0.789
gi|113648660	Os12g0147200	327	19.1	32	3	1.048	1.299	0.807
Energy production								
gi|113537902	Cytochrome b5 domain-containing protein-like	279	13.3	55.9	4	1.207	1.786	0.676
Lipid metabolism								
gi|113623031	Acyl-CoA-binding protein	200	13.5	54.9	2	1.660	2.026	0.819
Protein synthesis, folding and degradation								
gi|113549785	60S ribosomal protein L13a-4	85	31.1	6.3	1	0.541	1.081	0.500
gi|113624448	Cysteine proteinase 3 (Fragment)	182	48.9	13	3	1.177	1.663	0.708
gi|548770	60S ribosomal protein L3	64	61.3	6.7	2	0.169	0.238	0.710
gi|113649731	Ubiquitin-conjugating enzyme E2 variant 1C	238	19.5	29.5	2	0.734	1.031	0.712
gi|113535206	Dnak-type molecular chaperone Bip	4150	92.7	42.7	21	0.696	0.961	0.724
gi|556560	26S protease regulatory subunit 6A homolog	413	58.0	16.6	4	0.500	0.690	0.725
gi|113535132	RuBisCO large subunit-binding protein subunit beta, chloroplastic (Fragment)	791	80.6	20.1	4	0.566	0.777	0.728
gi|222642001	Putative UIP2 (SKP1-like 2, UFO-binding protein 2)	153	23.7	27.4	2	0.423	0.545	0.776
gi|75322635	Protein disulfide isomerase-like 2-3	289	60.2	15.6	5	0.817	1.018	0.803
gi|113579164	Luminal-binding protein 2/B-70/Bip 2	454	86.3	4.1	3	0.579	0.718	0.806
Redox homeostasis								
gi|113579377	Aldose reductase	222	44.4	27.7	6	1.183	1.575	0.751
gi|113550330	Putative oxidoreductase	97	42.0	9.9	2	0.789	1.030	0.766
gi|52076516	Thioredoxin O, mitochondrial	65	24.5	14.3	2	0.508	0.629	0.808
gi|516839	Catalase isozyme B	319	64.3	22.2	5	0.719	0.878	0.819
gi|119370643	Glutaredoxin-C8	398	18.8	44.9	6	0.680	0.824	0.825
gi|29367419	Peptide methionine sulfoxide reductase B5	265	21.3	29.8	3	1.188	1.437	0.827
Unknown pathway								
gi|284431762	Prolamin PROL 17D	356	18.6	35.9	1	0.435	1.319	0.330
gi|62733566	Os03g0723400	335	14.3	70.9	3	0.581	1.683	0.345
gi|306415963	Glycine-rich RNA-binding protein 1 (Fragment)	2043	17.0	41	1	0.496	1.351	0.367
gi|122222504	Protein SGT1 homolog	302	52.6	7.1	2	0.512	1.148	0.446
gi|113624371	Os08g0543600	98	104.8	3.7	2	0.625	1.182	0.529
gi|113536747	Os02g0576400	123	14.0	26.9	2	0.886	1.655	0.535
gi|113537457	Os02g0715400	298	11.9	32.7	2	0.848	1.440	0.589
gi|28564794	Putative uncharacterized protein P0534A03.117	258	25.8	24.4	3	0.792	1.342	0.590
gi|255672911	Vegetative cell wall protein gp1-like	108	66.4	8.1	3	0.979	1.430	0.685
gi|113578822	Ran-binding protein 3	82	63.4	4.9	2	1.220	1.719	0.710
gi|77556700	Os12g0626500	1606	16.3	38.6	3	1.019	1.396	0.730
gi|113537680	Annexin D7	144	42.1	13.4	4	0.747	0.951	0.785
gi|113533811	Putative calcium-binding protein	245	72.6	8.6	4	1.015	1.290	0.787
gi|18652814	Zinc finger protein ZAT7	102	19.7	8.8	1	1.165	1.420	0.820

Note: “-”, not detected.

In summary, we identified 113 proteins responsible for grain chalkiness formation. Of these, 52.1% proteins involve in carbohydrate metabolism, nitrogen metabolism including amino acid biosynthesis and protein synthesis, assembly, and degradation, and redox homeostasis.

### Proteins associated with carbohydrate metabolism

**Figure 6:**
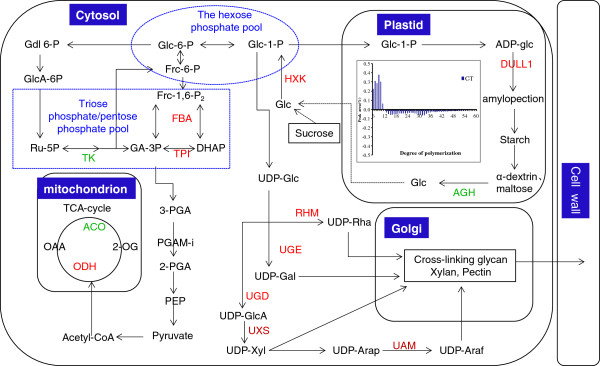
**Overview of differentially expressed proteins onto carbohydrate metabolism associated with rice grain chalkiness.** Up-regulated proteins are designated in red font and down-regulated proteins are designated in green font. **ACO**, aconitate hydratase; **AGH**, alpha-glucosidase; **Araf**, arabinofuranose; **Arap**, arabinopyranose; **DULL1**, putative starch synthase DULL1; **FBA**, fructose-bisphosphate aldolase; **Frc**, fructose; **GA-3P**, glyceradehycle 3-phosphate; **Gal**, galactose; **GalA**, galacturonic acid; **Gdl**, gluconolactone; **Glc**, glucose; **GlcA**, glucuronic acid; **HXK**, hexokinase; **OAA**, oxaloacetate; **ODH**, 2-oxoglutarate dehydrogenase; **P**, phosphate; P**EP**, phosphoenolpyruvate; **PGAM-i**, 2,3-bisphosphoglycerate independent phosphoglycerate mutase; **RHM**, rhamnose biosynthetic enzyme; **Rha**, rhmannose; **Ru-5P**, ribulose 5-phosphate; **TK**, transketolase; **TPI**, triosephosphate isomerase; **UAM**, UDP-arabinopyranose mutase; **UGD**, UDP-glucose 6-dehydrogenase; **UGE**, UDP-glucose 4-epimerase; **UXS**, UDP-glucuronic acid decarboxylase; **Xyl**, xylose; **2-OG**, 2-oxoglutarate; **2-PGA**, 2-phosphoglycerate; **3-PGA**, 3-phosphoglycerate.

Seventeen differentially expressed proteins associated with carbohydrate metabolism were identified. These proteins were metabolic enzymes directly involved in starch synthesis and hydrolysis, cell wall biogenesis, glycolysis, pentose phosphate pathway and TCA cycle. As shown in Figure 6, most of the enzymes identified are up-regulated, except for three enzymes in the TCA cycle (aconitate hydratase), pentose phosphate pathway (transketolase), and starch hydrolysis (α-glucosidase).

Notably, proteins involved in starch accumulation were promoted. The enzyme Dull1, which is responsible for the synthesis of long chain of amylopectin, was up-regulated. By contrast, the α-Glucosidase involved in starch hydrolysis was down-regulated. In addition, we identified five up-regulated enzymes related to cell wall synthesis, including rhamnose biosynthetic enzyme, UDP-glucose 4-epimerase, UDP-glucose 6-dehydrogenase, UDP-arabinopyranose mutase, and UDP-glucuronic acid decarboxylase. This finding indicates the potential role of cell wall synthesis in the formation of endosperm chalkiness.

### Proteins involved in protein synthesis, assembly and degradation

**Figure 7:**
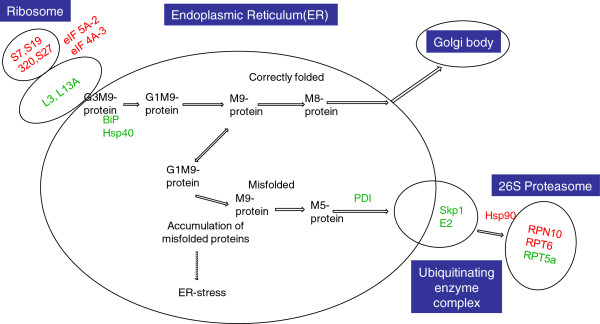
**Differentially expressed proteins associated with protein synthesis, folding, and degradation.** Up-regulated proteins are designated in red font and down-regulated proteins are designated in green font. **BiP**, dnak-type molecular chaperone BiP and Luminal-binding protein 2; **Hsp**, heat shock protein; **PDI**, protein disulfide isomerase like 2–3; **RPN10**, 26S proteasome non-ATPase regulatory subunit 10; **RPT5a**, 26S protease regulatory subunit 6A homolog; **Skp1**, putative UIP2 ( SKP1-like); **RPT6**, 26S proteasome regulatory particle triple-A ATPase subunit 6.

We obtained 31 proteins related to protein synthesis, folding and degradation (Figure 7). For protein synthesis, basic components of ribosome, mainly the 60S ribosomal proteins (L3 and L13a-4) were identified as being down-regulated, whereas 40S ribosomal proteins (S7, S19, S20, and S27) were identified as being up-regulated. In addition, eIF 4A-3 and eIF 5A-2 proteins were up-regulated. Four molecular chaperones were found to be down-regulated in the endoplasmic reticulum, including BiP1 (dnak-type molecular chaperone BiP), BiP2 (luminal-binding protein 2), PDIL 2–3 (protein disulfide isomerase-like 2–3), and Hsp40 (dnaJ homolog subfamily B member 11). Six proteins were identified in the ubiquitin/26S proteasome system, the major location for protein degradation. Among these proteins, the RPN10, RPT6, and Hsp 90 were up-regulated. However, the ubiquitin-conjugating enzyme E2, Skp1, and RPT5a were down-regulated. Previous studies showed that, knockout of PDIL genes like PDIL 1–1 can result in grain chalkiness [19], and mutant with over-expressed or knockdown of BiP genes displayed an opaque phenotype [7]. Thus the down-regulation of PDIL 2–3 and BiP proteins identified in this study could be responsible for the occurrence of endosperm chalkiness.

### Proteins related to redox homeostasis

**Figure 8:**
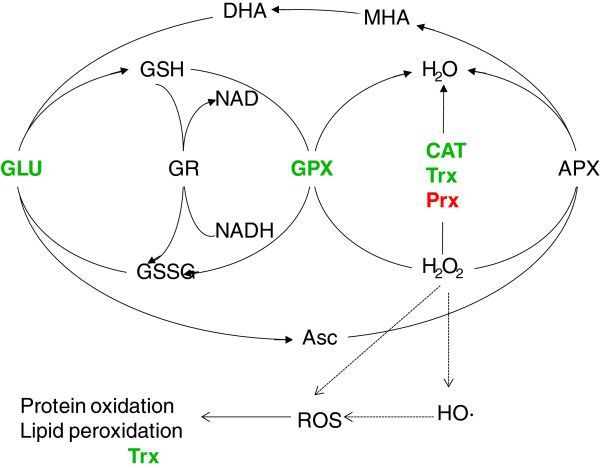
**Differentially expressed proteins involved in ROS-scavenging mechanism.** Up-regulated proteins are designated in red font and down-regulated proteins are designated in green font. **Asc**, ascorbate; **APX**, ascorbate peroxidase; **CAT**, catalase; **DHA**, dehydroascrobate; **MDA**, monodehydroascorbate; **GLU**, glutaredoxin; **GPX**, glutathione peroxidase; **GSH**, reduced glutathione; **GSSG**, oxidized glutathione; **ROS**, reactive oxygen species; **TRX**, thioredoxin; **Prx**, peroxidase.

The Asc/GSH cycle, consisting of glutathione, ascorbic acid and related metabolic enzymes, is an important part of ROS scavenging mechanism to reduced peroxides [20]. Liu *et al.*[5] reported that an imbalance in ROS concentrations in endosperm may contribute to the development of grain chalkiness, and glutathione-S-transferase, glyoxalase, lipoxygenase-5, and thioredoxin (TRX) were responsible for maintaining the homeostasis of ROS. We found four proteins involved in the ROS-scavenging mechanism of rice chalky endosperm (Figure 8). Among these, catalase isozyme B, thioredoxin O, glutaredoxin-C8 were down-regulated, whereas peroxidase 2 were up-regulated. Our findings agree with those of Liu *et al.*[5], suggesting a close relation between redox homeostasis and grain chalkiness.

### Additional proteins related to grain chalkiness

In addition to proteins involved in carbon and nitrogen metabolism, and redox homeostasis, proteins of five additional metabolic processes should be mentioned. (1) proteins associated with inorganic ion transport like protein CutA1 and sulfurtransferase. (2) signal transduction related proteins, such as calmodulin-2 and calmodulin-2 that mediates the control of a large number of enzymes, ion channels, and other proteins by Ca^2+^. (3) proteins involved in cell defense, such as late embryogenesis abundant protein Lea14-A, pathogenesis-related protein, and major pollen allergen Bet v 1-D/H. (4) the seed storage protein prolamin PPROL 17D. (5) proteins involved in other metabolisms except carbon and nitrogen, including lipid metabolism (non-specific lipid-transfer protein and Acyl-CoA-binding protein), coenzyme metabolism (C-1-tetrahydrofolate synthase), and secondary metabolites biosynthesis (4-hydroxy-3-methylbut-2-en-1-yl diphosphate synthase). Collectively, our results indicate that the formation of grain chalkiness is associated with diverse biological processes and multiple genetic pathways.

## Discussion

### The practical value of the comparison system based on the notched-belly mutant with white-belly

Grain chalkiness has been the major objective of rice breeding and cultivation. It is a rather complicated trait controlled both by genetic and environmental factors. Up to now, genomic, transcriptional, and proteomic approaches have been adopted to explore the genes or biochemical pathways responsible for chalkiness formation, by which our knowledge of grain chalkiness has been broadened [5,9,21]. Nevertheless, only few QTLs or genes have been identified and functionally analyzed. The molecular mechanisms of endosperm chalkiness are still imperfectly understood.

Proteomic method has been employed to elucidate the physiological foundation of grain chalkiness, and the effect of high temperature has been investigated extensively [9,10]. However, comparisons of protein expression in these studies were conducted either between varieties or between chalky grains and perfect grains for a given variety. These comparisons have limitations that it can not avoid the effect of genotype for grains between varieties and the effect of growing environment for grains of the same variety. Even if they are from the same panicle, the different position means different flowering date, which results in different meteorological conditions for grain filling.

We identified a notched-belly mutant with white-belly (DY1102) in 2010. This mutant has high percentage of notched-belly grain. The notched line is visible on the fifth day after anthesis, which enable us chasing the process of chalky tissue formation. Notably, the white-belly only occurs in the bottom part proximal to the embryo, with the upper half part being translucent. In addition, about 15% of the notched-belly grains do not have white-belly, which can be used as the control for comparisons. Using this mutant, we developed a novel comparison system, and thereby enzymes or biochemical pathways responsible for chalkiness formation were identified, including pathways of carbohydrate metabolism, protein synthesis, folding, and degradation, and ROS scavenging. These pathways confirmed the previous findings by other researches [5,8,9], and should be further examined in future studies on grain chalkiness.

It should be noted that some results of this study are contrary to previous studies, which may be associated with the materials used. Take cell wall synthesis as an example. Liu *et al.*[5] made a transcriptional comparison between a near-isogenic line CSSL50-1 with high chalkiness and its normal parental line Asominori for grain endosperm chalkiness. Their results showed that the enhancement of sucrose and starch synthesis in grains of CSSL 50–1 is at the cost of cell wall related nonstorage polysaccharides, suggesting the possible role of the disorders in carbohydrate metabolism in the formation of endosperm chalkiness. By contrast, our results revealed the promotion of starch synthesis is accompanied by the increased expressions of five enzymes involved in cell wall synthesis (Table 2). Interestingly, this result was obtained by comparisons between T1 vs T2 and C1 vs C2, using grains without white-belly as control, by which the effect of embryo was evaluated. However, if this control is not used, the comparison between C1 and C2 would result in an opposite conclusion that the five proteins were down-regulated in the chalky tissue. This example highlights the high value of DY1102-based comparison system to exploring the underlying mechanism of chalkiness formation.

### Carbon metabolism in relation to grain chalkiness

#### Starch synthesis

Amylopectin makes up 65–85% of starch weight. The major starch synthase (SS) isozymes responsible for amylopectin biosynthesis in rice endosperm are SSI, SSIIa, and SSIIIa. SSI generates chains with a DP 8–12 from short DP 6–7 chains [22]. By contrast, SSIIIa elongates the amylopectin B2–4 chains with DP >30 [23]. Proteomic analysis of this study showed that DULL1 (SSIIIa) was up-regulated. However, the increased expression of DULL1 did not cause increase in the ratio of long chains. As revealed by the results of HPSEC, in comparison with the translucent parts, the chalky part contained more short chain (DP ≤ 12) and less medium and long chain. Fujita *et al.*[24] reported that SSI or SSIIIa is indispensable for starch biosynthesis in rice endosperm, and these isozymes can strongly compensate for each other when the counterpart is lacking. Thus it is still unknown whether the increased level of short chain is the result of compensation from other SSs like SSI or not, which needs further investigation.

#### Cell wall synthesis

Cell walls make up a major part of the dry weight of plants, and at times of rapid growth, their synthesis may be a major drain on the resources of the cell, accounting for 30% or more of cellular carbohydrate metabolism. Five enzymes responsible for cell wall synthesis, including rhamnose biosynthetic enzyme, UDP-glucose 4-epimerase, UDP-glucose 6-dehydrogenase, UDP-arabinopyranose mutase, and UDP-glucuronic acid decarboxylase, were detected in this study. In addition, the five proteins were all up-regulated in the chalky endosperm of grains with white-belly. This finding disagrees with that of Liu *et al.*[5], which showed down-regulation of genes responsible for cell wall synthesis (two cellulose synthase genes) and up-regulation of genes for cell wall hydrolysis (α-L-arabinofuranosidase and α-D-xylosidase). This is partially due to the differences in: (1) materials used, as mentioned above; and (2) methodology employed between proteomics and transcriptome [5]. Nevertheless, the two studies collectively indicate the significance of the cell wall synthesis for the elucidation of chalkiness formation.

#### Starch degradation

In addition to starch synthesis, there is growing awareness of the role of starch hydrolysis in the occurrence of grain chalkiness. The following evidences support this: (1) SEM photos showed that many micro-pores occur on the surface of the compound granules. The pores are viewed as the evidence of α-amylase attack, indicating the involvement of starch-degrading enzymes in grain chalkiness [25,26]. (2) The level of glucose in opaque endosperm was markedly high as compared with the corresponding translucent parts of perfect grains, indicating that amylolytic enzymes exist and work in the opaque parts of chalky grains [27]. (3) The mRNA expression of *Amy1A*, *Amy1C*, *Amy3D*, and *Amy3E* genes, as well as α-amylase activity, increased under high temperature stress, suggesting a relation of starch degradation by high-temperature induced α-amylase to the formation of grain chalkiness [28,29]. α-Glucosidase degrades the product of α-amylase and β-amylase, maltose, short chain glucans, and maltosaccharides or limit dextrins, to glucose. Our proteomic analysis showed that it was down-regulated in the chalky part, confirming the relation between starch hydrolysis and chalkiness formation.

On the other hand, Ishimaru *et al.*[30] reported that the α-amylase mRNA was not detected in the chalky tissues during grain filling, suggesting that starch degradation by α-amylase not be the cause of the formation of chalky grain. However, these authors argued that the formation of chalkiness through the starch degradation α-amylase may have occurred at the later stage [30]. In this study, aldose reductase (AR) was identified as being down-regulated in the chalky tissue. It catalyzes D-glucose converted to D-sorbitol, and serves a functional role in the desiccation tolerance processes at late stage of grain filling [31]. Therefore, temporal expression pattern of proteins should be fully investigated in order to evaluate individual contribution of starch degrading enzymes or late expressed proteins like AR to chalkiness formation.

### Role of protein accumulation in grain chalkiness formation

Proteins account for about 8% of the rice endosperm’s weight, filling the space between starch granules. The role of proteins in the formation of grain chalkiness has gained increasing awareness. For example, results of Lin *et al.*[9] suggested the relation between protein (small heat shock proteins) accumulation and chalkiness formation under high temperature. In this study, we found that proteins belonging to protein folding and degradation were differentially expressed between the chalky and translucent part of the notched-belly grains, indicating the important role of proteins in grain chalkiness formation.

#### Protein folding

Protein must fold into a precise three dimensional structure to carry out their biological function, which is regulated by a group of proteins referred to as molecular chaperones. Chaperones fall into two major groups. The first group, the Hsp70 family such as BiP1 and BiP2 maintains polypeptides in an unfolded state. The second family is the chaperonins, including protein disulfide isomerase (PDI), promote proper polypeptide folding. There is evidence that changes in BiP protein levels induce ER stress, and BiP overexpression results in an opaque phenotype in the whole endosperm of rice [32]. PDI-like (PDIL) proteins are members of a multigene family belonging to the thioredoxin (TRX) superfamily [19]. The failure to express PDIL genes results in a floury endosperm and an endoplasmic reticulum stress response in rice. Han *et al.*[21] reported a mutant T3612 with a deletion in a gene encoding a protein disulphide isomerase-like enzyme (PDIL1-1) produces small grains with a floury endosperm. In addition, the absence of PDIL1-1 is associated with endoplasmic reticulum stress in the endosperm, which is likely to underlie the formation of the floury endosperm in the mutant. In this study, down-regulation of molecular chaperones including BiP1, BiP2, and PDIL2-3 were detected in the chalky tissues, confirming the relation between protein folding and chalkiness formation. In review of findings of other studies which indicate a strong correlation between the expression of PDIL and BiP and grain chalkiness formation, we suggest that these chaperones should be emphasized in the studies on chalky formation.

#### Protein degradation

Protein degradation plays many important physiological role in the cell, removing abnormal proteins, facilitating the recycling of amino acids, and regulating protein activity by eliminating molecules that are no longer needed. One major proteolytic pathway in eukaryotes involves the Ubiquitin/26S Proteasome System that utilizes the post-translational modification of proteins by ubiquitin. The conjugation of ubiquitin to a protein is carried out by a ubiquinating enzyme complex, consisting of a ubiquitin-activating enzyme (E1), ubiquitin-conjugating enzyme (E2), and a ubiquitin-ligating enzyme (E3). Among them, E3 and E2 are considered to play a crucial role in the specificity of ubiquitination. Six proteins were detected in the ubiquitin/26S proteasome system, with Skp1 (E3) and E2 being down-regulated, indicating the involvement of protein degradation in the grain chalkiness. In addition, Zhang *et al.*[33] reported that mutants in *OsVPS22* gene responsible for ubiquitin-mediated degradation of membrane proteins had a chalky endosperm in the grain, supporting the hypothesis that protein degradation has implication in grain chalkiness formation.

### The importance of balance between C and N metabolism in future studies on grain chalkiness

So far, the majority of studies with respect to grain chalkiness have been focused on starch, relating its accumulation or degradation to the formation of chalky tissue [28,34]. Our previous work showed that the insufficient accumulation of protein bodies that do not completely fill the air spaces between starch granules may be an explanation for chalkiness occurrence, as was also reported by Del Rosario *et al.*[35]. Therefore, both starch and proteins are equally important for the formation of grain chalkiness.

In rice grain, starch and protein are the products of carbon (C) and nitrogen (N) metabolism. During the rice seed development, sugars and amino acids transported from source organs are allocated to C and N metabolism to produce starch and proteins in precise quantities and ratios, which requires coordinated expression profiles of genes involved in synthesis of starch and storage [36]. Disorder or breakdown of the balances between C and N may be the possible causes of chalkiness formation.

So far, the mechanism by which plants receive and transduce signals relating to their C and N status and subsequently regulating C and N metabolism remains unresolved. In the future studies on grain chalkiness, more attention should be paid to three enzymes, which perform regulating function in grain C and N metabolism. (1) Hexokinase (HXK). Recent studies in plants have unveiled sugar sensing and signaling systems mediated by hexokinase as a glucose sensor in a hexokinase-independent way [37]. (2) Aspartate aminotransferase (AAT). It catalyzes the reversible transfer of the amino group from aspartate to α-ketoglutarate, yielding oxaloacetate and glutamate. The ability of AAT to interconvert these important C and N compounds places it in a key position to regenerate plant metabolism. For example, using enzymes including AAT and AS, plants direct assimilated nitrogen into inert asparagines, which has a higher N: C ratio than glutamine and therefore can transport and store nitrogen more efficiently when carbon skeletons are limiting. (3) Thioredoxin (TRX). It was reported that TRX *h* targets at proteins involved in C and N metabolism, like sucrose synthase, phosphoglycerate kinase, alanine aminotransferase, and BiP. Increased TRX *h* expression resulted in aberrant phenotypes, such as chalky and shriveled features of rice grains under high temperature [38]. This is partially associated with the breakdown of the balance between C and N metabolism, leading to the abnormal biosynthesis of storage materials.

In this study, the above three proteins, HXK1, AAT, and TRX were detected to be differentially expressed between the chalky endosperm and its counterpart translucent endosperm. An extensive investigation of proteins or genes regulating C and N metabolism, in particular HXK1, AAT, and TRX, should extend our knowledge of the mechanisms with respect to chalkiness formation.

## Conclusions

Using iTRAQ and the novel comparison system, we compared the chalky part with the translucent part of a notched-belly mutant with white-belly. Consistent with previous studies, our comparative proteomic analysis reveals immense complexity of the mechanism underlying rice grain chalkiness. Notably, nearly half of the identified proteins are involved in several central metabolic or regulatory pathways including carbohydrate metabolism, protein synthesis, folding and degradation, and ROS metabolism. However, key proteins of interest, in particular those involved in cell wall synthesis and protein folding, need to be confirmed using other methods like Western blotting. This study provides a valuable proteomic resource for the characterization of grain quality pathways at molecular and biochemical levels. Further refining of DY1102 as genetic material will help eventually clone and engineer the major genes related to the occurrence of rice grain chalkiness.

### Availability of supporting data

The mass spectrometry proteomics data have been deposited to the ProteomeXchange Consortium [1] via the PRIDE partner repository with the dataset identifier PXD001030.

## Abbreviations

AAT: Aspartate aminotransferase; AR: Aldose reductase; AS: Asparagine synthetase; BiP: Binding protein; DP: Degree of polymerization; EMS: Ethyl methane sulfonate; FBA: Fructose-1, 6-bisphosphate aldose; GO: Gene Ontology; HPLC: High performance liquid chromatography; HPSEC: High performance size exclusion chromatography; HSP: Heat shock protein; HXK: Hexokinase; iTRAQ: isobaric tags for relative and absolute quantification; LC-MS/MS: High-performance liquid chromatography with tandem mass spectrometry; PDI: Protein disulphide isomerase; PDIL: Protein disulphide isomerase-like; PPDK: Pyruvate orthophosphate dikinase; QTLs: Quantitative trait loci; ROS: Reactive oxygen species; SEM: Scanning electron microscope; sHSP: small heat shock protein; TPI: Triose-phosphate isomerase; TRX: thioredoxin; UPLC: Ultra performance liquid chromatography.

## Competing interests

The authors declare that they have no competing interests.

## Authors’ contributions

ZL, XZ, and ZL conceived and designed the experiments; GL, ST, and SW performed part of the experiments; ZL, YD, and ZL prepared the manuscript. All authors have read and approved the final manuscript.

## Supplementary Material

Additional file 1 List of all proteins identified and quantified for iTRAQ experiment, including the protein and peptide output files. Click here for file
